# Optimization of Wire Arc Additive Manufacturing (WAAM) Process for the Production of Mechanical Components Using a CNC Machine

**DOI:** 10.3390/ma16010017

**Published:** 2022-12-20

**Authors:** Anamaria Feier, Ioan Buta, Cosmina Florica, Lucian Blaga

**Affiliations:** 1 Department of Materials and Manufacturing Engineering, Mechanical Faculty, Polytechnic University Timisoara, Bl. Mihai Viteazu No. 1, 300222 Timisoara, Romania; 2 Department of Computers and Exact Sciences, Faculty of Engineering, Ioan Slavici University, Paunescu Podeanu Street No. 144, 300569 Timisoara, Romania; 3 Mechanical Faculty, Polytechnic University Timisoara, Bl. Mihai Viteazu No. 1, 300222 Timisoara, Romania; 4 Department Solid State Materials Processing, Institute for Materials Mechanics, Helmholtz Zentrum Hereon, 21502 Geesthacht, Germany

**Keywords:** additive manufacturing, automotive, manufacturing costs, wire arc additive manufacturing

## Abstract

The paper presents a CNC component manufacturing process using the WAAM process. The study depicts all the execution steps of a component from the CAD drawing, deposition procedure (technological parameters, times, layers, etc.), examination, and economic calculation. The manufacturing of this component using WAAM is more advantageous given the fact that the execution time and delivery are significantly shorter, mainly when a single piece is required and also when discussing the raw material used, usually expensive titanium alloys. For example, for Ti-6AI-V used in the aircraft industry, for which the material price is about 90 Euro/kg, the costs for obtaining a given component using the WAAM process will be about 497 Euro/piece compared to 1657 Euro/piece when using another manufacturing process, as it is shown in this paper. In conclusion, additive manufacturing can easily become a feasible solution for several industrial applications when it replaces a classic manufacturing process of a single component or replacement products, even simple-shaped.

## 1. Introduction

Additive manufacturing (AM) is a production method based on the addition of layered material, thus obtaining functional products. Additive fabrication or 3D printing is very popular nowadays because it covers a series of processes designed to produce parts or assemblies from different types of materials. Basically, 3D printing turns a three-dimensional design into a physical object. The common element of all 3D printing technologies is the way of obtaining these components—overlapping layers of material that lead to the final shape of the printed object [1].

In 3D-printing processes, several materials can be used, some of which we expect—plastic, metals, ceramics, or even concrete, but also surprisingly paper or edible materials such as chocolate. Regarding the 3D printing of metals, in 1926, Ralph Baker (USA) patented the use of the electric arc as a heat source to generate 3D objects by layer by layer-by-layer deposition [2,3,4].

**Figure 1:**
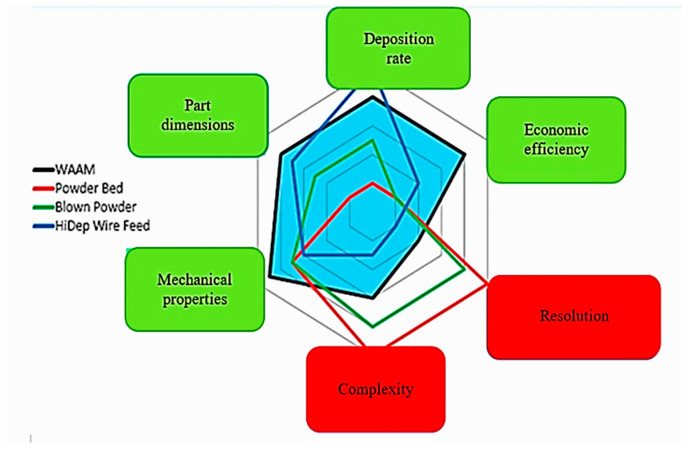
Comparison of advantages and disadvantages between different methods of AM [5].

As shown in Figure 1, the WAAM—Wire Arc Additive Manufacturing—process offers advantages over other processes in terms of the mechanical properties obtained, the dimensions that can be achieved, the deposition rate, and low costs. The disadvantages of this process are the limitations related to the complexity of the shapes that can be achieved as well as the low accuracy that involves further machining [5].

Wire Arc Additive Manufacturing (WAAM) is a manufacturing process with arc energy used to melt and deposit the filler material in successive layers to form parts with homogeneous structure [6,7]. Using this process, different components or entire structures such as the 3D-printed pedestrian bridge designed by Joris Laarman and built by Dutch robotics company MX3D can be obtained [8].

Unlike the most common AM processes in cases where metal powders are used, the WAAM principle is melting metal wire using the electric arc as a heat source. The process is controlled by a robotic arm and the work piece is built on a substrate material (a support plate), and, in the end, the workpiece/component can be cut once finished or the substrate material can be embedded in the finished product. The wire, when melted, is deposited in the form of a welded seam on the substrate. As the successive seams are deposited, they create a layer of metal material. The process is then repeated, layer by layer, until the metal piece is completed [5,9,10].

The research in wire arc additive manufacturing (WAAM) is being driven by the need to further improve the manufacturing efficiency of engineering structures. Using WAAM, components very near to net shape preforms can be produced without the need for complex tooling or dies, thus showing potential for significant cost and lead-time reductions, increased material efficiency, improved mechanical performance, and reduced inventory and logistics costs. The process was patented in 1920 and is probably the oldest but least talked about of the range of additive manufacturing (AM) processes (commonly known as 3D printing) [8,11,12]. By using wire as feedstock, the basic process has been used to perform local repairs on damaged or worn components, and to manufacture round components and pressure vessels in the past. The advantage of computer-aided design and manufacturing (CAD/CAM) software has made Additive Manufacturing, in general, possible, with this process being an area of significant development in recent years, with a resolution of approximately 1 mm and deposition rate between 1 and 10 kg/hour or more (depending on type of arc source) [13,14,15].

**Figure 2:**
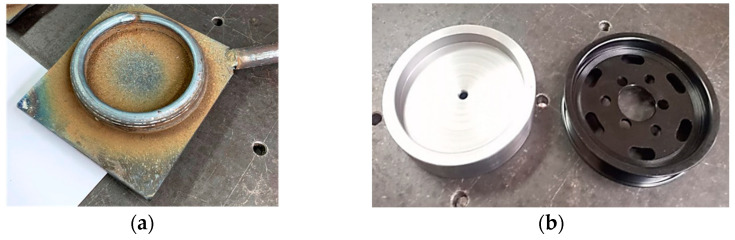
Exploratory study—automotive component using the WAAM—of first trials to obtain finished products with this process, prior (**a**), post-finishing (**b**).

The present study highlights the possibility of producing some components using the WAAM (Wire Arc Additive Manufacturing) process. In Figure 2, an example of an exploratory automotive component manufactured and designed using WAAM is presented by the same working collective that also developed this CNC component from the present research. A support plate was used to manufacture the product; after optimizing the deposition parameters, the piece was obtained in a short time, with the unprocessed specimen (only the deposited area) obtained in 54 min. Subsequent machining on the latter took an additional 2–3 h [16,17]. The component obtained in Figure 2 was produced using the same principle as the component that is described in detail in this study from the procedure of deposition to the part of examination and cost.

## 2. Materials and Methods

This section presents the procedure of obtaining the piece/component. The piece was obtained by deposition layer-by-layer using the WAAM process. The material used was S355J0, and the filler material was G 42 4 M21 3Si1 with the following mechanical characteristics:

- Yield strength of 380 N/mm^2^ (depending on shielding gas);
- Tensile strength of 490 N/mm^2^ (depending on shielding gas).

The shielding gas used was Ar85%-CO_2_15%. Technological parameters of the working procedure of wire arc additive manufacturing (WAAM) are presented in Table 1.

The minimum required width of the deposited material should be 10 mm to provide sufficient material as machining addition. Initially, a trial was conducted for producing the piece in one single pass/layer, but the width of the deposited material, 8 mm, was not sufficient. Consequentially, the amperage (Is) was increased from 130 A to 180 A. It was observed that the first 2 layers reached the desired width, but the excessive residual heat accumulated in the workpiece or the too short of a cooling time led to an excessive melting of the layers deposited in the 3rd pass.

Taking these aspects into consideration, the following was established:

- The final part was produced with 2 passes/layer with a gap between layers of 4 mm;
- Amperage of 130 A;
- 15 L/min gas flow.

The infrastructure used in the experimental tests is the following:

1. Welding source: Sincosald Nova Plus 500 e inverter with Feeder 4R NSP wire feeder.
2. Shielding gas used: Argon 85% + CO_2_ 15% mixture (Corgon 18-Linde).
3. Yaskawa robot model MH 24—for welding gun operation.
4. Yaskawa positioning device model DK 250—capacity of 2500 kg used to fix and rotate the substrate material.
5. Protection, control, and robot control panel and positioning device.

**Figure 3:**
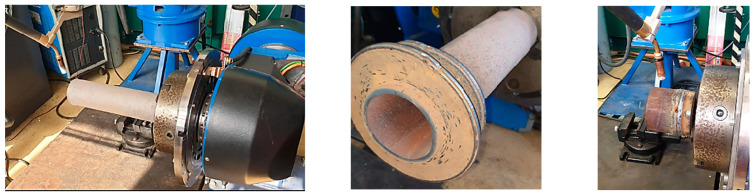
Steps from the working procedure of wire arc additive manufacturing.

The piece was obtained by depositing layer-by-layer on a pipe, as can be seen in Figure 3.

### 2.1. Residual Stresses and Strains

To reduce the effects of stress and deformations, but also to guarantee the necessary processing addition, the workpiece was made by deposition/welding in a pilgrim’s step in such a way as to allow sufficient cooling of the part between 2 consecutive layers.

During preliminary tests, it was established that a temperature of 300 °C at the beginning of consecutive layer deposition guarantees optimal deposition conditions. It was also checked that the temperature variation between the deposition of two consecutive layers was not greater than 5 °C. Temperature control during deposition was carried out using a Testo thermal imaging camera [18,19,20].

To monitor the temperatures in an efficient and consistent way, a reference point was chosen where temperatures were measured at the beginning of the weld/deposition seam and at the end of the weld/deposition seam on both welded sectors/diameters. For this purpose, the temperature measurement point was set as the end of the weld seam [21,22,23,24].

**Figure 4:**
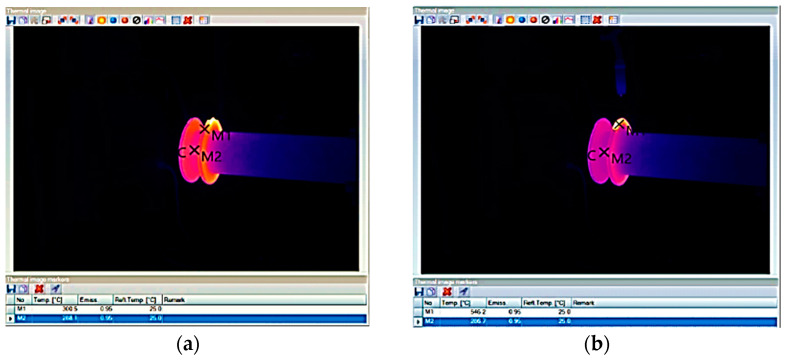
Thermography of the weld seam at the diameter of 170 mm, layer no. 10. Before welding (**a**) and after welding (**b**).

**Figure 5:**
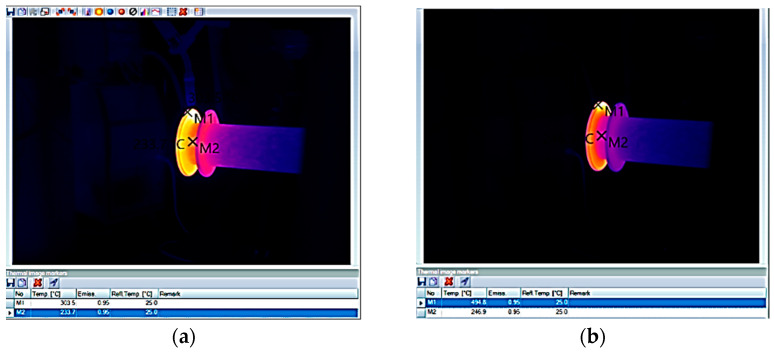
Thermography of the weld seam at the diameter of 195 mm, layer no. 15. Before welding (**a**) and after welding (**b**).

As seen in Figure 4 and Figure 5, the temperature of the layer deposited at the end of the deposition was located around 500–540 °C, and before starting the deposition of the successive layer, a cooling time of the part down to 300 °C was required. Therefore, a cooling time between 2 and 2.5 min was applied.

By analyzing the data, some preliminary conclusions can be drawn:

- The efficiency of the process for a single piece was only 45%. From a productive point of view, there were apparently massive losses. Nonetheless, the inefficiency of the process can be significantly diminished if two components were produced in parallel or by implementing an efficient cooling system.
- Deposition rate: it was necessary to perform 4 seams/layer instead of 2. If two parts had been produced in parallel, the amperage (Is) could have been higher because there would have been enough cooling time between the deposits.
- The maximum temperature of the deposited layer, i.e., 300 °C, before the start of the welding/deposition of the consecutive layer proved to be correct because, following the controls carried out, the deformation of the substrate material was less than 0.5 mm—measured at the inner diameter of the tubular substrate blank [25].

### 2.2. Preparation of the Piece for Turning

#### 2.2.1. Cutting

The length of the tubular blank used as a substrate material was greater than the length required to manufacture the flange. Accordingly, excess material was removed with the help of an angle grinder.

#### 2.2.2. Turning

**Figure 6:**
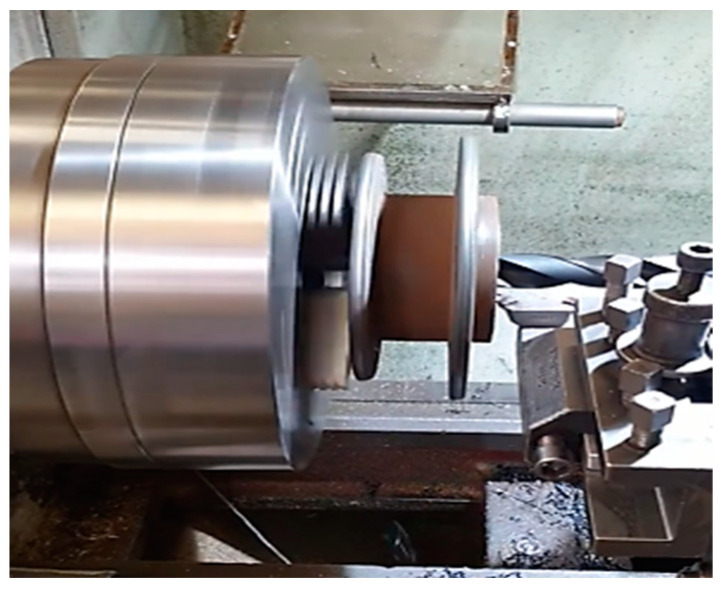
Excess material removals from the substrate.

Before the start of the turning operation, difficulties were encountered in centering the part in the lathe due to deformations of the inner diameter of the pipe (substrate material). These deformations were noticed before starting the welding/deposition process but were neglected because the clamping was carried out on the outer diameter that showed no deformations. Subsequently, in the turning phase, it was noticed that this deformation created positioning problems, so it was decided to build elements that would help center the piece in the lathe at the final turning phase. A phase of this procedure is shown in Figure 6 [26,27].

#### 2.2.3. Blasting and Preparing the Part for 3D Scanning

**Figure 7:**
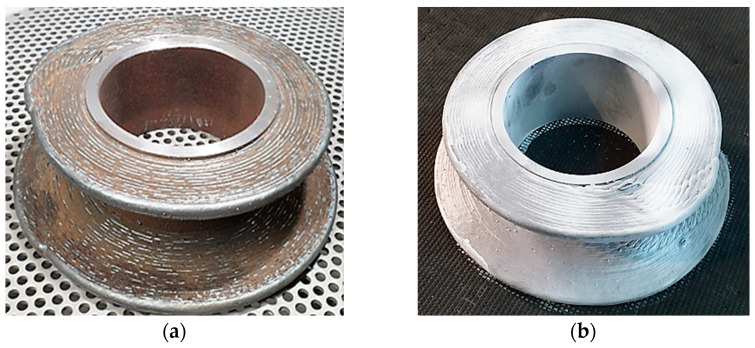
Sandblasting of part. (**a**) Before sandblasting; (**b**) after sandblasting.

As shown in Figure 7a, a small number of oxides and silicates were formed on the surface of the work piece. These deposits were removed by sandblasting with Corindom to prepare the part for 3D scanning.

**Figure 8:**
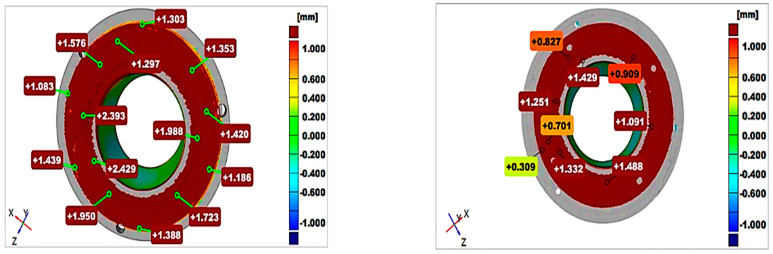

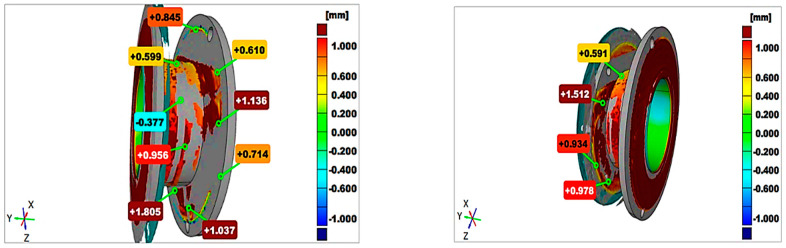
Three-dimensional Part Scan. Red areas represent the scanned part that are bigger than the 3D part, Green areas represent the scanned part is equal with 3D part, Blue areas are showing that the scanned part is smaller than the 3D part.

Three-dimensional scanning (Figure 8)—The purpose of this procedure was to check the deviations in the shape and quantity of the processing addition material of the part/component obtained in comparison with the 3D-designed model of the component [28].

The 3D scanning revealed that the component built on the substrate material was not perpendicular to the axis of the part, but this deviation still allowed a functional finished product to be obtained.

#### 2.2.4. Shaping the Piece

**Figure 9:**
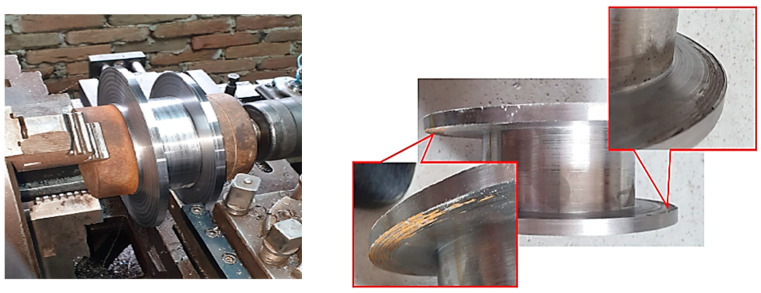
Shaping the piece.

A conventional manual lathe was used for shaping. As mentioned above, it was necessary to build elements for centering and fixing the part in the lathe to minimize the eccentricity caused by the deformation inside the pipe and the slight lack of coaxiality between the axis of rotation of the tubular blank and the axis of rotation of the positioning device in the welding phase. This led to the effect of deposition in the walls of the component with a slight deviation in perpendicularity, as visible in Figure 9.

It can be seen from the images below that the piece was dimensionally compliant.

#### 2.2.5. Correction of the Part

**Figure 10:**
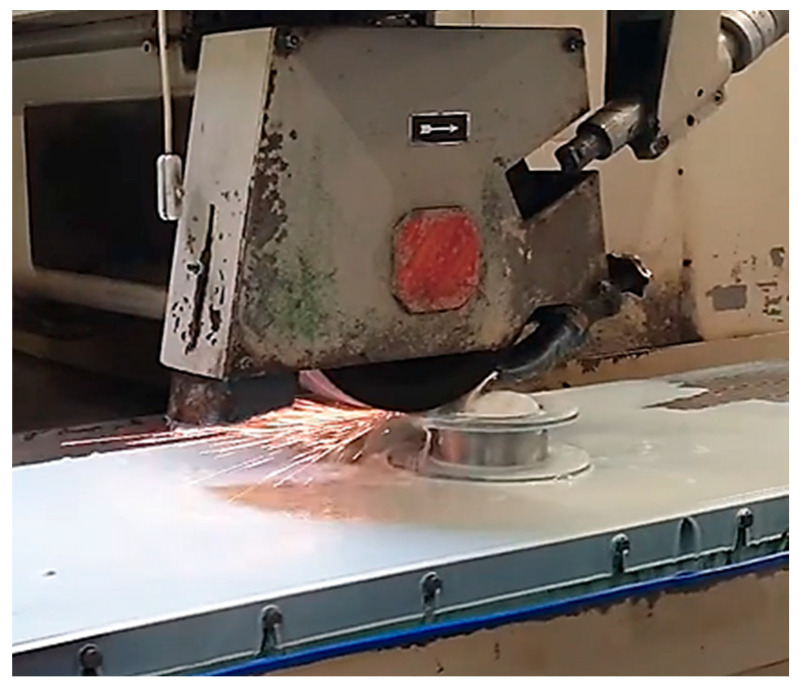
Grinding machine.

The proper functioning of the produced component depends on the parallelism of the tooth planes of lying. To guarantee this, it was considered necessary to carry out its flat rectification operation. The rectification was performed on a specialized machine for flat surfaces. The procedure can be seen in Figure 10.

**Figure 11:**
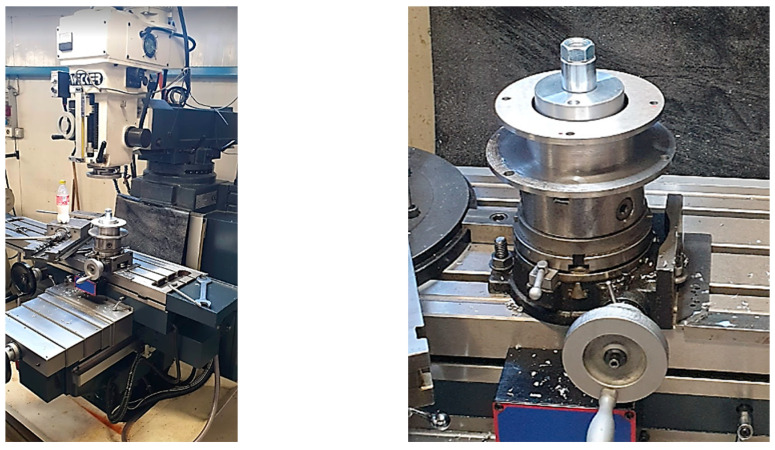
Fixing the part on the milling machine table.

After rectification, the parallelism of the 2 settlement planes was checked, resulting in a deviation of less than 0.005 mm. Execution of holes for the piece fastening are presented in Figure 11.

## 3. Results and Examination

### 3.1. Visual Control

During the deposition of the piece/component, the quality of the deposition was controlled visually, layer by layer. No unconformity was noticed during the construction of the work piece by welding/deposition. All weld/deposition seams had a homogeneous, regular appearance, without unevenness, pores, cracks, or other defects.

**Figure 12:**
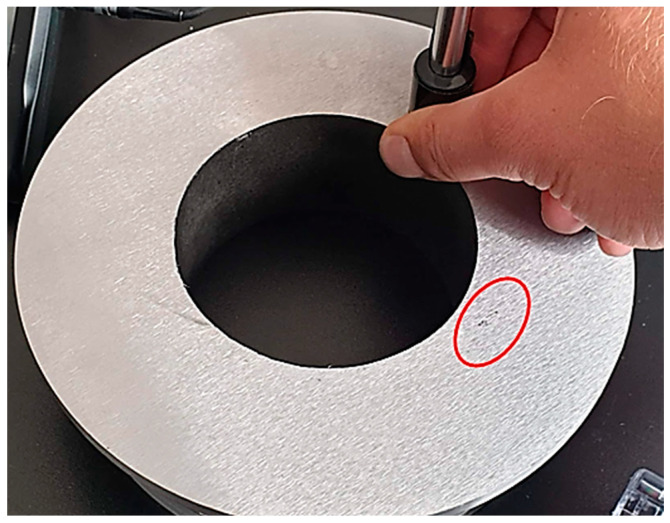
Presence of pores at the root layer, area inside of the red circle.

After milling, the presence of pores was observed to be located at the root level, on a length of 9 mm. Observed pores had a diameter of less than 1 mm, as can be seen in Figure 12.

Possible causes of pores in the layers:

*The surface of the substrate material could be contaminated with oily residues*—although the substrate material was mechanically cleaned to remove the oxides, it is possible that oily residues remained on the surface of the part because the degreasing operation was not performed before the start of the welding/deposition.

*Problems with the feed of the shielding gas*—possible accumulation of moisture/condensation in the gas circuit. Considering the occurrence of imperfections, it is possible that the shielding gas line was self-cleaned as the welding/deposition process proceeded.

*Too low welding/deposition speed*—the porosities were discovered only in the area of the first seam deposited at both ends of the piece, i.e., at the base of the 170 mm disc, as well as at the base of the 195 mm disc.

*High gas flow*—as the welding/deposition was robotized and the fact that more layer-by-layer deposition was carried out, an increased gas flow was chosen (15 L/min) close to the maximum limit, to obtain a better protection and stability of the arc. However, this hypothesis does not explain the fact that imperfections were located only in the first weld seam. The study needs to be more in-depth with the next components that will be obtained by this process.

### 3.2. Dimensional Checks

The piece was measured using the sliding caliper. The parallelism between the two settlement planes was measured using a micrometric colon placed on a granite plate.

**Figure 13:**
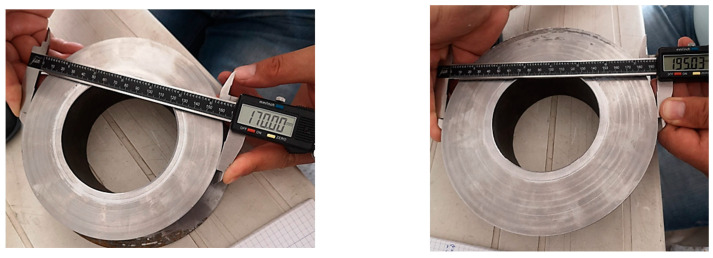
Dimensional control of the external diameters obtained after machining.

As can be seen from the images below (Figure 13), the piece was dimensionally compliant. The measured values were within the tolerance limits set in the execution drawing.

For the piece to serve the purpose for which it was built (fixing the universal to the positioner table), it is mandatory to have a perfect parallelism between the two settlement planes. For this purpose, an processing addition was left after turning, which was later removed in the phase of flat grinding [29,30,31].

### 3.3. Hardness Control

**Figure 14:**
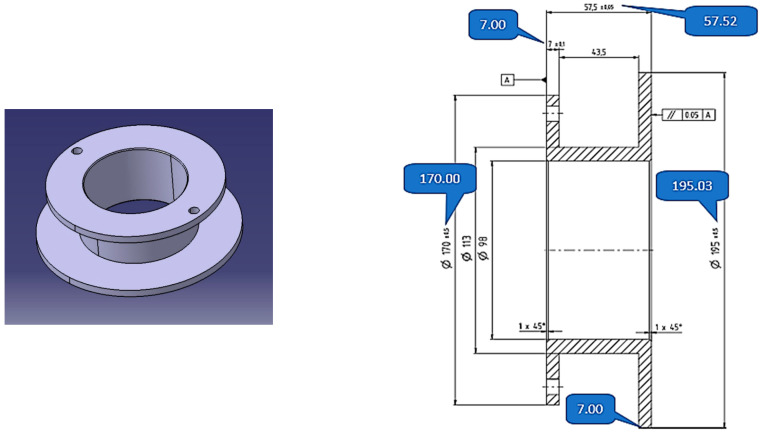
Measured part hardness values in different zones.

After flat grinding, the hardness of the part was checked using a portable instrument. The results of the tests are presented in Table 2 with details in Figure 14.

**Figure 15:**
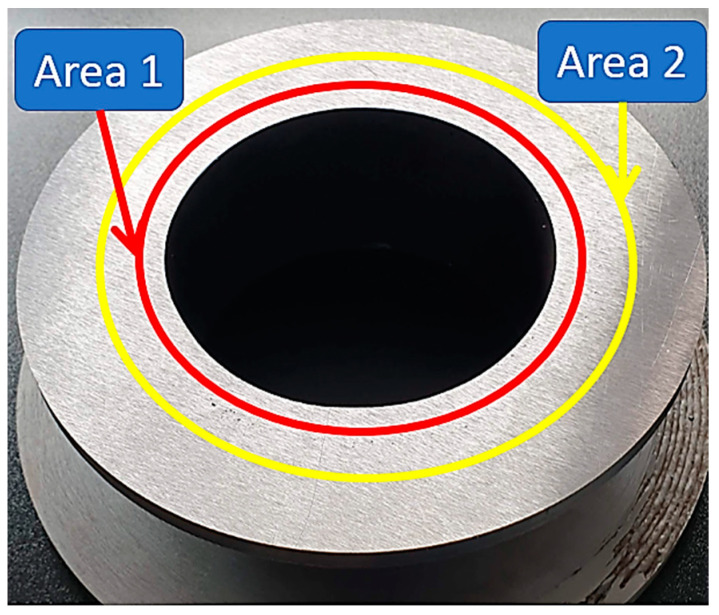
Highlighting the areas where hardness has been checked. Red circle: first deposition layer, Yellow circle: in the center of the successive deposited layers.

The hardness test was carried out in two distinct areas; namely, as can be seen in Figure 15, the two areas were very well explained (graphical and with text explanation).

Zone 1 is in the closeness of the first deposition layer. In this area, the hardness was checked because that zone was the dilution zone between the substrate material and the first deposition layers.

It can be observed from Table 3 that the average hardness measured in zone 1 was 153 HB. This hardness corresponds to the hardness values indicated by the manufacturer of the substrate material (S 355J0).

Zone 2 is the middle zone of the piece. As expected, the hardness of the part in the area achieved by deposition, 128 HB, was lower than in the dilution area due to the lower concentration of carbon in the addition material (0.08% vs. 0.20% for the S335J0) as well as due to the low cooling speed of the weld seams.

**Figure 16:**
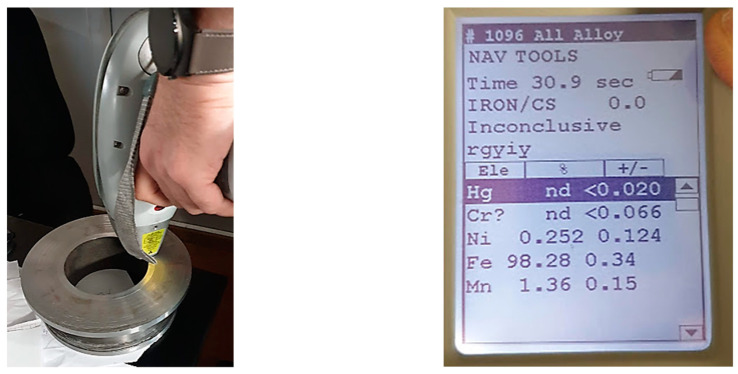
Comparison of results for filler wire. Chemical composition of the deposited material.

The chemical composition was also checked and compared with the results of the chemical composition declared by the manufacturer for the filler wire. The procedure can be observed in Figure 16.

To verify the chemical composition of the material deposited layer by layer, a portable device branded Niton™ XL3t XRF was used. No values were recorded outside the tolerances specified by the wire manufacturer.

**Figure 17:**
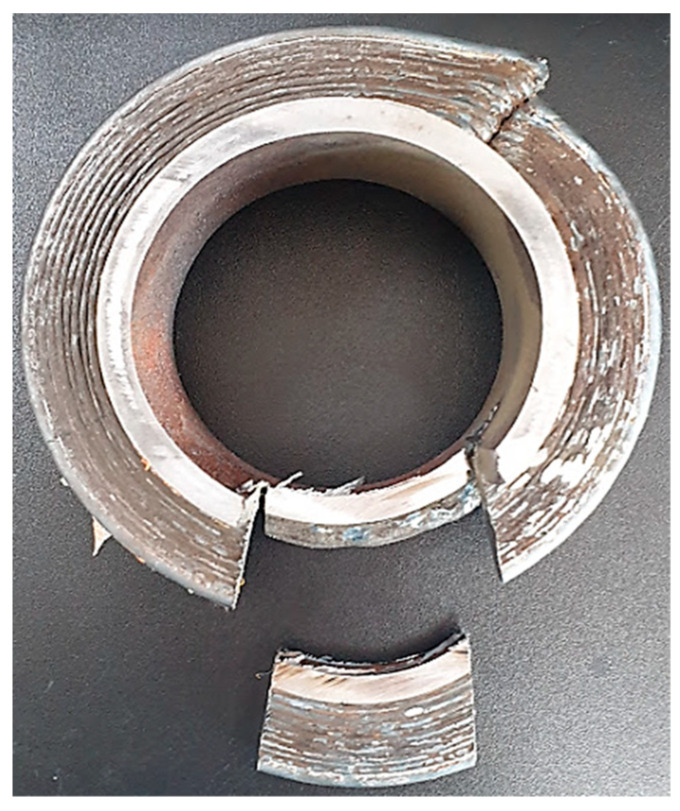
Sample sampler from the champion piece.

The section of examination was subject to microscopic analysis to observe the microstructure of the deposition material. The first step was to make the test-piece, namely the sample of the champion piece presented in Figure 17.

**Figure 18:**
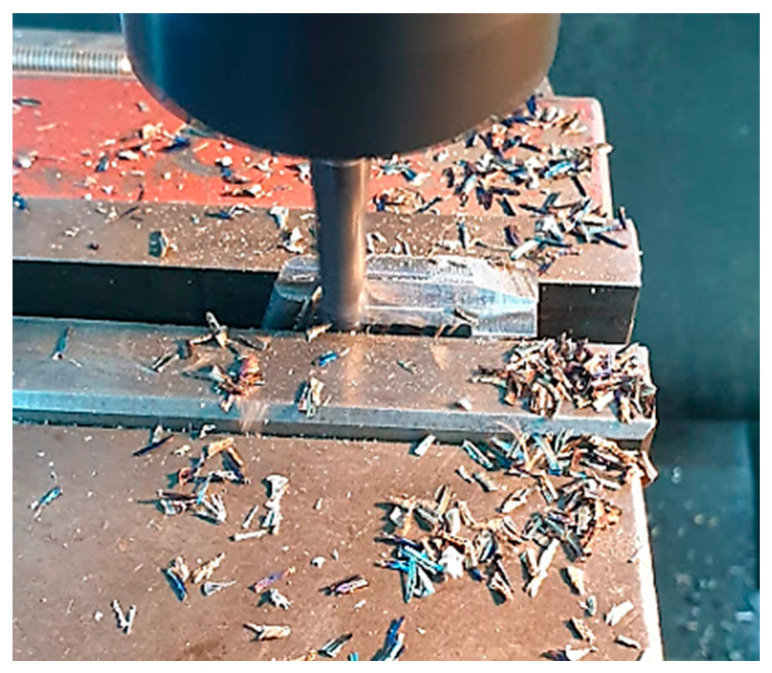
Milling the test tube.

**Figure 19:**
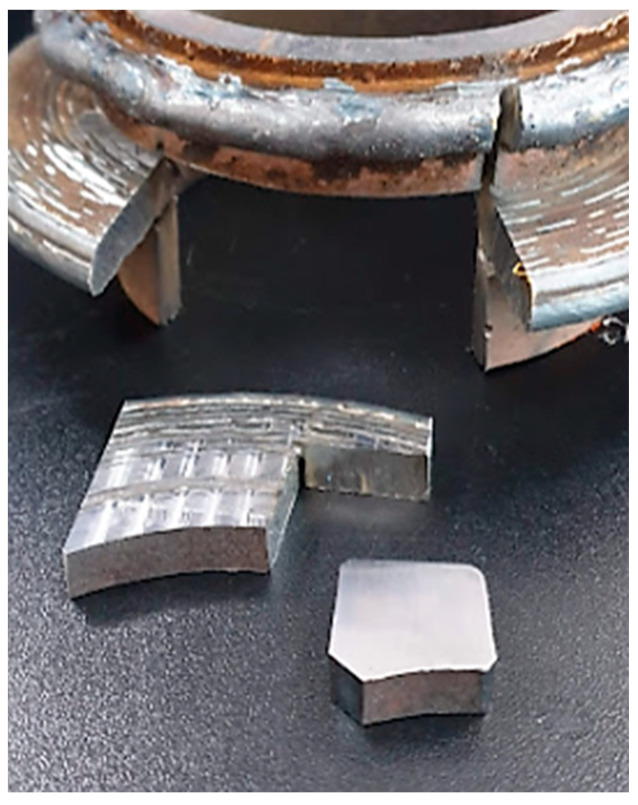
Preparation for resin incorporation.

Another step was milling of the test tube presented in Figure 18, a step that was needed to prepare the sample for microscopic tests. The following step was to cut and adjust the sample for embedding in resin (step presented in Figure 19).

Preparation for resin embedding (Figure 18).

**Figure 20:**
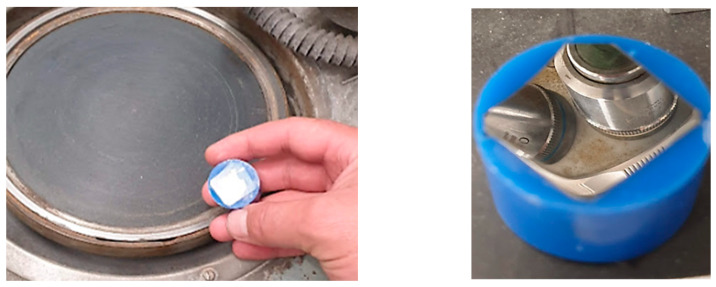
Embedding in resin and grinding/polishing.

Embedding in resin and grinding/polishing (Figure 20).

The test sample was progressively sanded, using abrasive paper of different grain sizes: 180, 600, 1200, 2500, and 4000, and after, it was polished using 1 μm diamond paste [32,33].

### 3.4. Metallographic Attack

The attack operation aimed to highlight the microscopic structure. The polished surface was attacked with the appropriate reactivity that selectively dissolves or stains the various constituents present, making it possible for them to be distinguished from each other.

The attack was carried out either by immersion or by swabbing the surface of the sample with a cotton wool piece.

**Figure 21:**
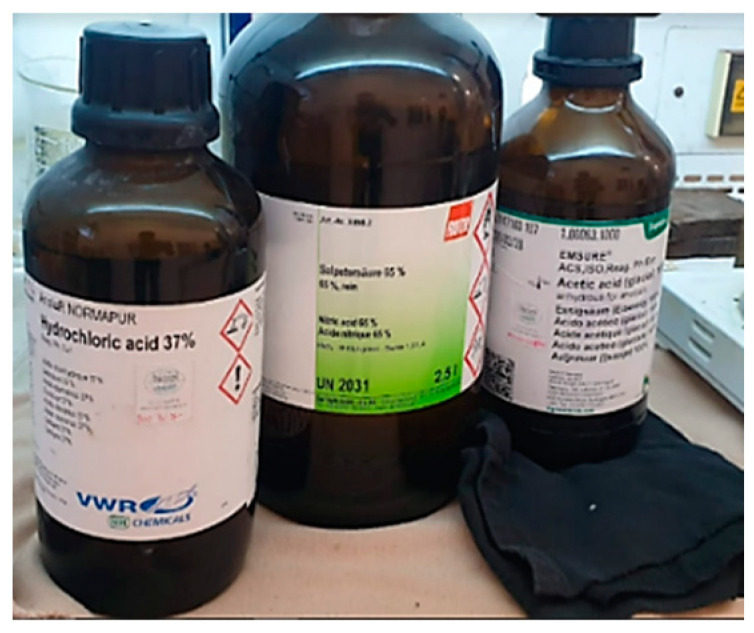
Reagent used to perform metallographic attack.

Metallographic reagents differ depending on the nature of the material and the purpose of the attack (STAS 4203- 74). For this sample, for the reagent consisting of HNO_3_, HCl, and CH_3_COOH in the figure below (Figure 21) [34,35,36], all components of the reagent can be seen.

**Figure 22:**
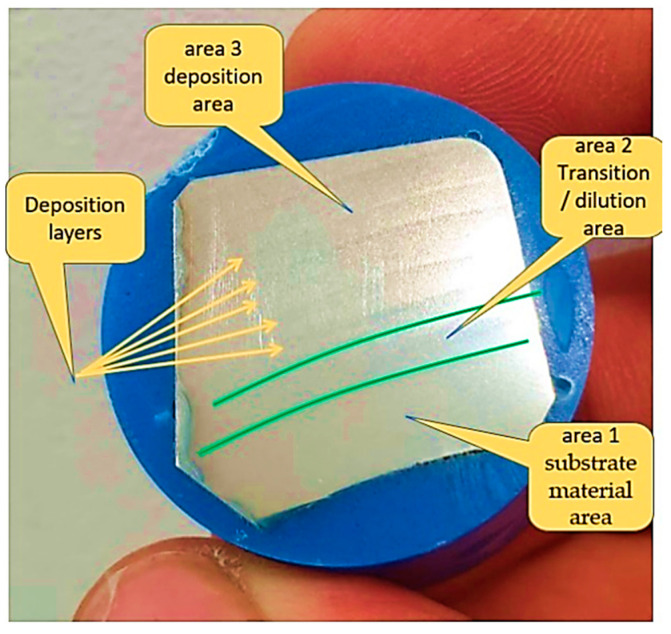
Highlighting microscopically analyzed areas.

The sample was attacked when the prepared surface lost its brilliancy and became slightly matte (Figure 22). Too intense an attack distorts the structure. After the metallographic attack, the sample was washed with water then with alcohol and dried by pressing against a filter paper or under a stream of warm air after that was examined with metallographic microscope.

### 3.5. Microscopic Examination—Interpretation

The test tube was analyzed using a microscope connected to the PC. The test sample was extracted from the champion piece in such a way that on its surface, there were three distinct zones (Figure 22), namely:

Area 1—substrate material area.

Area 2—transition zone (dilution).

Area 3—the area deposited layer by layer, highlighting the deposited layers.

Figure 22 presents very well all areas of the sample, and each zone is very distinctive. The deposited layers can be seen very well even in a simple picture without microscopic analysis (yellow outlined arrows shown in Figure 22).

**Figure 23:**
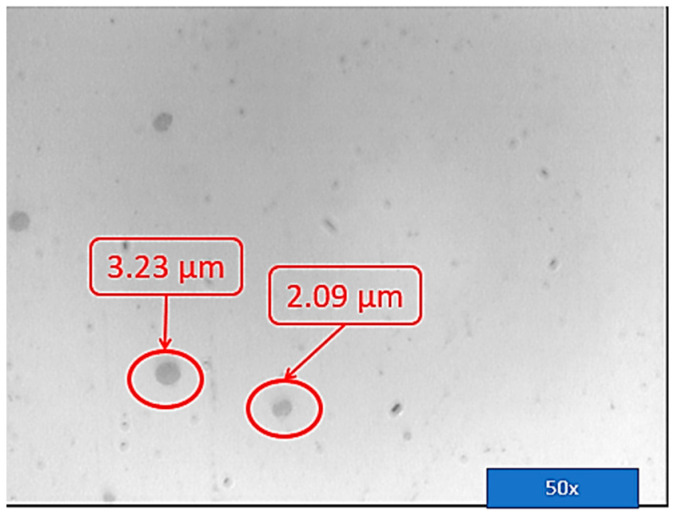
Microscopic porosities. Dimensional control of the porosities.

Before the metallographic attack, a microscopic examination of the three areas mentioned above was carried out. No unconformities were identified in zone 1 and zone 2. In zone 3, in the deposited material layer by layer, the presence of very small pores was observed. The diameter of the porosities found in the additional material was less than 3 μm (Figure 23) (these are not serious defects but are defects within the limits recommended by the standards, and above in the article, possible cases of occurrence are explained) [37].

### 3.6. Substrate/Basic Material Area 1

The substrate material chosen was a tubular steel blank S355J0 with the mechanical characteristics shown in Table 3. The filler material used was Bohler G 42 4 M21 3Si1 electrode wire with a diameter of 1 mm, which has the characteristics shown in Table 4 [38,39].

The wire electrode was an uncoated wire designed for low spatter and arc welding with high stability for a wide range of welding parameters.

ECOspark series uncoated wires feature high feed rates, high arc stability, and a low formation of oxides and silicates on the weld surface. This filler material is recommended for fully mechanized welding.

**Figure 24:**
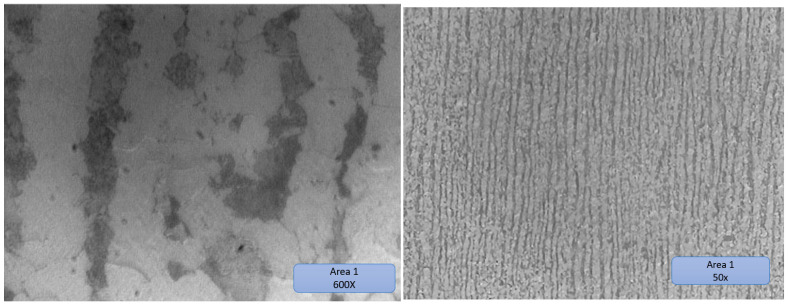
Substrate material area 1.

The substrate has a ferritic–pearlitic rolling structure, typical of cold-rolled materials, to which the parallax blades (dark in color) alternate with the ferritic grains (light in color) presented in Figure 24.

**Figure 25:**
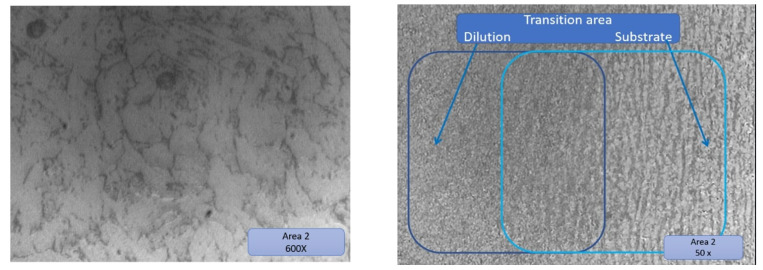
Substrate material area 2, with dilution, substrate, and their overlapped transition area marked in rectangles.

**Figure 26:**
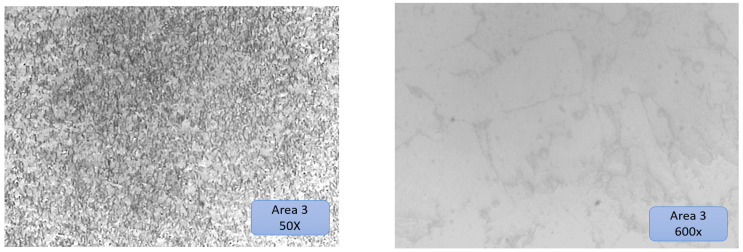
Substrate material area 3.

Substrate material areas 2 and 3 are presented in Figure 25 and Figure 26, respectively.

The structure of zone 2 was a solidification structure, presenting a mixture of ferritic grains with a low volume of pearlitic elements with a random orientation. The higher share of ferrite is most likely due to the high speed when cooling the metal deposited in the first layer on the cold substrate, which partially suppressed the formation of perlite.

The subsequent thermal cycles in the analyzed area were not sufficient to restore the ferrite/pearlite ratio in the probable layer from the low peak temperatures or the short heating time in the analyzed area [40,41].

Zone 3 is a typical normalization zone, resulting from the thermal effect of successive layers on previously deposited layers with small equiaxial grains.

### 3.7. Economic Calculation

Ludwig von Mises addresses the problem of economic calculation—necessarily monetary—in his work Human Action: “Our civilization is inseparably linked to the methods of economic calculation. It would perish if we abandoned this so precious tool of action” [42]. Each step in the sphere of entrepreneurial activities shall be subject to verification by means of monetary calculation.

**Figure 27:**
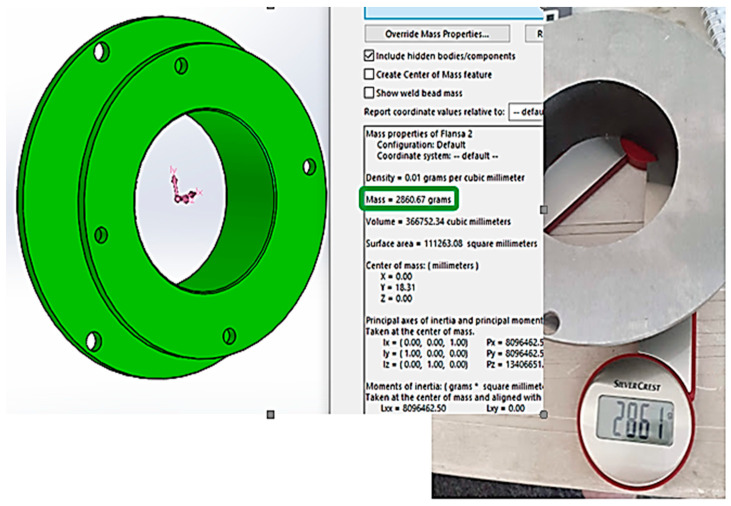
Comparison between the calculated part weight and the weight obtained.

As shown in Figure 27, the total weight of the component produced was 2861 g, a weight very close to the weight calculated by the Solid Works design software; in SolidWorks, 2860.67 g was obtained.

## 4. Manufacturing Costs Using WAAM Materials

Table 4 shows the detailing of the costs of the raw material used to produce the component as well the calculation of the rate of use of the material. As can be seen, the total weight of the raw part was 4185 g, and after being needed to machine the workpiece, the weight dropped to 2861 g. Thus, for the flange produced using the WAAM process, the loss rate of the material was 42.21%, the equivalent of 4.56 Euro losses/piece produced.

### 4.1. Raw Material Costs Using the Classic CNC Cutting Process

As shown in Table 5, the weight of the raw material required was 17.153 g for the realization of the component that will have the final weight of 2.861 g. The result was a loss of 83.32% of the semi-finished product, i.e., the equivalent of 20.72 Euro losses/piece produced.

Comparison of the costs of workmanship WAAM–CNC cutting:

WAAM workmanship—The time required to achieve the component by the WAAM process was 202 min (it also includes the time needed to cool between two consecutive deposits). The time needed to obtain the finished piece, using a classic lathe, was 60 min. Thus, the total time to obtain the finished product was 262 min. It is, however, important to mention that the production process through the WAAM was not optimized in the sense to an unproductive time generated by the fact that the component had to cool down enough to be able to deposit the layer successively. The WAAM process can be reduced by half because the geometry and welding times for the developed workpiece are sufficient to produce two workpieces simultaneously. Thus, an optimized WAAM process would take about 160 min to produce a finished component.

**Figure 28:**
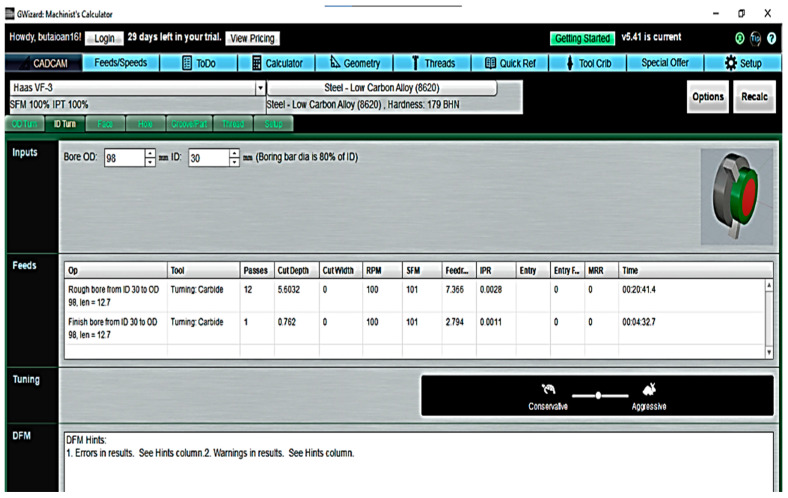
Software for CNC simulation turning the component.

CNC workmanship—For the comparison of manufacturing times using a CNC lathe, the GWizzard software Version number: 5.41, creator: CNCCookbook, location: Aptos, CA 95003 USA. (CNC Cookbook^®^) was used to simulate the time required to obtain the finished product (Figure 28).

Table 6 shows a detailing of the time required to perform the machining phases by cutting to obtain the finished part. Thus, the resulting total time was 151 min.

### 4.2. WAAM Total Cost Comparison—CNC Cutting

Table 7 synthesizes the comparative costs between the optimized WAAM process, the optimized WAAM process, and the conventional CNC turning process for component execution. As can be seen, the optimized WAAM process is slightly more expensive than the classic CNC process due to the waiting times for cooling the part, but if the part is produced with optimized WAAM, the production costs decrease significantly, i.e., −33%.

However, a very important aspect remains to be mentioned: when producing the component and when making economic calculations, the price of the raw material was 1.45 Euro/kg for massive S355J0 and 3.52 Euro/kg for the filler material, so the impact of the waste of raw materials at the CNC milling is not very important.

If, hypothetically, the component were made of a titanium alloy, for example, Ti-6Al-V used in the aeronautical industry, where the cost of the material is about 90 Euros/kg, the workpiece manufactured by the WAAM process would have a cost of around 497 Euro/piece compared to 1657 Euro/piece in conventional CNC manufacturing, resulting in a reduction of 72% WAAM manufacturing costs (Table 8).

## 5. Conclusions

The novelty of this study refers to the options a manufacturer can consider in order to produce a component, including small series, and gives them an idea of a more advantageous option using an innovative manufacturing process (WAAM) that is cost- and time-effective, compared to the other classic manufacturing processes. For example, to obtain a product for a small series of products if a CNC component is needed, and this piece usually is obtained by casting, this study demonstrates that it can be obtained in a justified time and with mechanical characteristics equivalent or even better than a cast component.

Depending on the specific situation, the following conclusions can be drawn:

- Regarding the economic aspect—the WAAM process is not recommended for the construction of parts with a simple geometry, because the classical manufacturing processes are much better optimized and adapted in this respect.
- Regarding the efficiency of the WAAM process in the case of the product presented in this paper, for a single workpiece, it was only 45%, and from a production point of view, the difference was represented as losses. The inefficiency of the process can be significantly diminished if two parts were produced in parallel or by implementing an efficient cooling system.
- In the case of deposition rate for the product developed in this study case: it was necessary to perform four layers instead of two. If two parts had been produced in parallel, the welding current could have been higher because there would have been enough cooling time between the deposits
- In terms of stresses and deformations, the situation resulted from parameters, and the maximum underpass temperature of the deposited layer, i.e., 300 °C, proved to be correct because following the controls carried out, the deformation of the substrate material was less than 0.5 mm—measured at the inner diameter of the tubular substrate blank.
- Regarding the resolution for the product developed in this study case, the quality of the surfaces of the parts obtained by WAAM was not very high, as further processing was required, but the quantity of material deposited in excess was good compared to the other advantages that this process brings.
- In terms of slicing, there are several pieces of software on the market that provides the interface between the CAD model and the robot’s operating program, but due to the geometric complexity of the parts, the diversity of materials, and the numerous final uses of the finished products, there are real difficulties in correctly programming welding cycles that may differ from layer to layer within the same part.

In the deposited material layer by layer, the presence of very small pores was observed but the diameter of the porosities found in the addition material was less than 3 μm. The quality of the part was higher, the part obtained by layer-by-layer deposition using the WAAM process, and there was a negligible porosity induced in the manufacturing process, which was distinguished by homogeneity of the deposited material.

In terms of hardness of the part in the area achieved by deposition(128 HB), the hardness was lower than in the dilu-tion area due to the lower concentration of carbon in the filler material (0.08% vs. 0.20% for the S335J0), as well as due to the low cooling speed of the weld seams.

Another conclusion of this study is the importance of correct programming of the robot when moving on height. The robot arm, or welding gun, must have an axis of vertical movement perpendicular to the axis of rotation of the workpiece for perpendicular deposition of the walls-component on the substrate surface. A lack of this perpendicularity may, as with coaxially, leads to flatness defects.

The time required to achieve the component by the WAAM process was 202 min. The time needed to obtain the finished product, using a classic lathe, was 60 min. Thus, the total time to obtain the finished product was 262 min. The WAAM process can be optimized in terms of times by producing two parts simultaneously. An optimized WAAM process would take about 160 min to obtain a finished CNC component [43].

A significant point in the production of the part and in the economic calculations is that the price of the raw material was 1.45 Euro/kg for S355J0 steel and 3.52 Euro/kg for the filler material, so the impact of raw material waste in CNC milling is not very important, but if the part were made of a titanium alloy, e.g., Ti-6Al-V used in the aircraft industry where the material cost is about 90 Euro/kg, the part obtained by the WAAM process would cost about 497 Euro/piece compared to 1657 Euro/piece in conventional CNC manufacturing, resulting in a −72% reduction in manufacturing costs by the WAAM process.

This study can be continued and divided in several directions as follows:

- Studying the possibility of producing the flange by executing only two weld seams per layer instead of the four current seams as well.
- Studying the impact on the mechanical properties of the part.
- Using a forced cooling system with cooled air at low temperatures (−20 °C, −30 °C) and studying the effects of forced cooling on the mechanical properties of the part.

## Figures and Tables

**Table 1:** Technological parameters of working procedure of wire arc additive manufacturing.

Technological Parameters	Value
Polarity	CC+
Amperage Is (A)	125
U_a_ operation voltage (V)	21.8
Rate of welding (m/min)	5.4
Gas flow Ar85%-CO_2_15% (L/min)	15
Medium linear energy (J/cm)	3096
Length of the free end (mm)	9
Positioning rotational speed (°/s)	4.93

**Table 2:** Hardness tests.

Nr.crt.	Test 1 (HB)	Test 2 (HB)	Test 3 (HB)	Test 4 (HB)	Test 5 (HB)	Average (HB)
Zone 1	155	152	155	154	152	153
Zone 2	126	127	129	130	132	128

**Table 3:** Mechanical characteristics and average of hardness tests.

Mechanical Properties	Value
Charge	S355J0
Rm (N/mm^2^) (SR EN 10025-2)	510–680
R_eH_ (N/mm^2^) (SR EN 10025-2)	355
HB (manufacturer)	154–208
HB (zone 1)	153
HB (zone 2)	128
R_eH_ of solid wire (N/mm^2^)—from manufacturer	380
Rm of solid wire (N/mm^2^)—from manufacturer	490

**Table 4:** Detailing the material costs for the WAAM process and the percentage of material use.

Basic Material	Before Processing	After Processing
Length (mm)	60.0	57.5
External diameter (mm)	113.0	113
Internal diameter (mm)	98	98
Density (gr/cm^3^)	7.8	7.8
Surface (mm^2^)	2485.8	2485.8
Volume (cm^3^)	1491.47	1429.33
Layer weight (gr)	1163.35	1114.97
Piece weight	4185	2861
Material weight of filler	3021.65	1746.13
Quantity of material removed after grinding (gr)	1276	
Percentage of material use (%)	57.79%	
Percentage of material loss after processing (%)	42.21%	
Electrode wire price G 42 M 21 3Sil (Euro/kg)	3.52	
Cost wire electrode/piece (Euro)	10.64	6.15
Loss of filler material/piece	−4.49	
material price S355J0 (Euro/kg)	1.45	
Cost of material/piece (Euro)	1.69	1.62
Loss of material/piece	−0.07	
Total material losses (Euro)	−4.56	

**Table 5:** Raw material cost and percentage material use for component construction using conventional CNC process.

Characteristics	Semi-Manufactured Article	Finished Article
Length (mm)	70.0	57.5
External diameter (mm)	200.0	113
Interior diameter (mm)	0	98
Density (gr/cm^3^)	7.8	7.8
Surface (mm^2^)	31,415.9	2485.8
Volume (cm^3^)	21,991.15	1429.33
Weight (gr)	17.153	1114.97
Amount of material removed after milling (gr)	14,292	
Percentage of material use (%)	16.68%	
Percentage of material loss after processing (%)	83.32%	
Prefabricated price (Euro/kg)	1.45	
Semi manufactured cost/piece (Euro)	24.87	4.15
Loss of filler material (Euro/piece)	−20.72	

**Table 6:** Simulation of CNC lathe cutting times.

	Phase	Operation	Ø (mm)	Length (mm)	Times (hh:mm:ss)
Clamping 1	Face turning	Rough turn	200	3.23	0:12:45
		Turn finish	200	0.76	0:15:45
	outer diameter cutting	Rough turn	195.762	7	0:00:33
		Turn finish	195	7	0:00:54
		Rough turn	170.762	7	0:01:39
		Turn finish	170	7	0:00:47
		Rough turn	113.762	43.5	0:24:28
		Turn finish	113	43.5	0:03:13
	Inside diameter machining	Drilling	30	80	0:07:10
		Reaming	98.76	80	0:20:41
		Finish ream	98	80	0:04:32
Clamping 2	Face turning	Rough turn	200	3.23	0:12:45
		Turn finish	200	0.76	0:15:45
Clamping 3	Hole execution	Front drilling 1 Ø 10.5 × 3	10.5	7	0:15:00
Clamping 4	Hole execution	Front drilling 1 Ø 8.5 × 4	8.5	7	0:15:00
		**Effective time to complete the piece:**			**2:30:57**

**Table 7:** Comparison of WAAM-CNC component manufacturing costs.

Comparative Evaluation of Manufacturing Costs WAAM vs. CNC			
Cost	Process WAAM Non-Optimized	Process WAAM Optimized	Classic Process (CNC)
Cost of raw material/material (Euro)	12.32	12.32	24.87
Total production time (min) with WAAM * non-optimized	202	100	151
Total machining time after WAAM	60	60	0
Hourly rate (Euro)	30	30	45
Manpower cost (Euro/hour)	131	80	113
**Total production cost (Euro)**	**143**	**92**	**138**

* WAAM optimized = refers to the fabrication of 2 parts in parallel to eliminate/reduce the waiting time for cooling of the part before starting the welding of the successive layer.

**Table 8:** Comparison of costs for piece/component of Ti-6Al-V.

Comparative Evaluation of Manufacturing Costs WAAM vs. CNC			
Cost	Process WAAM Non-Optimized	Process WAAM Optimized	Classic Process (CNC)
Cost of raw material/material (Euro)	376.65	376.65	1543.78
Total production time (min) with WAAM * non-optimized	202	100	151
Total machining time after WAAM	60	60	0
Hourly rate (Euro)	30	30	45
Manpower cost (Euro/hour)	131	80	113
**Total production cost (Euro)**	**508**	**457**	**1657**

* WAAM optimized = refers to the fabrication of 2 parts in parallel to eliminate/reduce the waiting time for cooling of the part before starting the welding of the successive layer.

## Data Availability

The data presented in this study are available on request from the corresponding author.
